# To Medical Publishers, Medical Associations, and Physicians

**Published:** 1872-02

**Authors:** John Stainback Wilson

**Affiliations:** Librarian and Curator, Atlanta Academy of Medicine


### TO MEDICAL PUBLISHERS, MEDICAL ASSOCIATIONS, AND PHYSICIANS.

The Atlanta Academy of Medicine having taken steps to establish a library and museum, the undersigned solicits, in behalf of the Academy, contributions of books, journals and transactions from publishers and medical associations, and pathological specimens from physicians. Medical publications will be submitted to the numerous members of the Academy, and will be noticed in the ATLANTA MEDICAL AND SURGICAL JOURNAL. Pathological specimens will be presented to the Academy and preserved in the museum, with the names of the donors. Such specimens are specially solicited, and will be highly prized.

All contributions may be sent by express, at the expense of the Academy, and should be addressed, *in full*, to

JOHN STAINBACK WILSON, M.D.,
*Librarian and Curator, Atlanta Academy of Medicine.*

